# Clinical benefit of continuing crizotinib therapy after initial disease progression in Chinese patients with advanced ALK-rearranged non-small-cell lung cancer

**DOI:** 10.18632/oncotarget.15892

**Published:** 2017-03-04

**Authors:** Xiangchan Hong, Qi Chen, Lingyu Ding, Ying Liang, Ningning Zhou, Wenfeng Fang, Xinru Chen, Haiying Wu

**Affiliations:** ^1^ Department of Medical Oncology, Sun Yat-Sen University Cancer Center, State Key Laboratory of Oncology in South China, Guangzhou, China; ^2^ Department of Oncology, The First Affiliated Hospital of Clinical Medicine of Guangdong Pharmaceutical University, Guangzhou, China; ^3^ Department of Medical Oncology, Hangzhou Cancer Hospital, Hangzhou, China

**Keywords:** non-small-cell lung cancer, anaplastic lymphoma kinase, crizotinib, treatment beyond disease progression, progression-free survival

## Abstract

**Purpose:**

Although most patients with *ALK*-positive non?small-cell lung cancer (NSCLC) who benefit from treatment with crizotinib ultimately develop progressive disease (PD), continuing crizotinb beyond the initial PD (CBPD) in these patients may be beneficial. In this study, we investigated whether Chinese patients with advanced *ALK*-positive NSCLC benefit from CBPD, and whether any factors are predictive of a longer post-initial progression-free survival time (PFS2).

**Materials and Methods:**

Data on 33 patients with ALK-positive NSCLC who achieved disease control with crizotinib were analyzed retrospectively. The impact of continued crizotinib therapy on the patients’ PFS2 time was assessed after adjusting for potential confounding factors.

**Results:**

With initial crizotinib therapy, the objective response rate (ORR) and median PFS time (PFS1) in the 33 patients were 63.6% and 8.6 months, respectively. With continued crizotinib therapy after documentation of PD, the median PFS2 for all 33 patients was 16 weeks, and in those with CNS progression but systemic disease control it was 30 weeks. Patients who received local therapy after disease progression had a significantly longer PFS2 compared with those who did not (*P* = 0.039). Multivariable Cox regression analysis showed that the PFS1 with initial crizotinib treatment and local therapy were independent predictors of PFS2.

**Discussion:**

This study provides further evidence of the benefit of continuing crizotinib therapy in Chinese patients with progressive ALK-positive NSCLC. Patients with a longer PFS1 and those who received local brain therapy would have a longer period of continuing crizotinib.

## INTRODUCTION

Lung cancer with a both high morbidity and mortality [1], is actually a heterogeneous disease in different patients as various driven-gene been identified [2–5]. Certain patients (e.g., those with adenocarcinoma histology, *EGFR* wild type, non/light-smokers, younger age) have rates of *ALK* rearrangements that approach 30%, and this group could benefit from the use of *ALK*-inhibitor treatment with crizotinib, a small molecule, multi-targeted tyrosine kinase inhibitor (TKI) [6]. A phase 1 study of crizotinib has shown it to have a clinical marked effect in the treatment of advanced non–small-cell lung cancer (NSCLC) patients with *ALK-EML4* rearrangement and other *ALK* gene rearrangements, with an objective response rate (ORR) of 60.8% and a progression-free survival (PFS) time of 9.7 months [7]. More recently, the PROFILE 1014 study in *ALK*-positive lung cancer patients demonstrated that crizotinib can increase PFS and ORR in comparison with first-line platinum-based agents [8]. On the strength of the available evidence, the US Food and Drug Administration (FDA) approved crizotinib for the treatment of NSCLC patients harboring *ALK* rearrangements.

Although patients with ALK-positive NSCLC may benefit from crizotinib, most ultimately develop progressive disease (PD). The mechanism of acquired resistance is thought to be due to an original gene alteration or activation of a signaling bypass pathway. CNS progression, which mostly results from the limited cerebrospinal fluid (CSF) penetration of crizotinib, is commonly seen in patients whose systemic disease is controlled. A retrospective analysis of the clinical benefits of continuing crizotinb beyond initial PD (CBPD) concluded that a longer overall survival was achieved in patients with advanced ALK-positive NSCLC [9]. Consequently, the National Comprehensive Cancer Network (NCCN) clinical guideline now recommends the resumption of crizotinb therapy when patients with disease progression do not have multiple systemic symptomatic lesions.

Because of the potential benefits of continued crizotinib therapy and the fact that second-generation ALK-inhibitors such as ceritinib or alectinib have not yet been approved by the Chinese FDA, we conducted a retrospective study to document our experience with CBPD therapy in Chinese patients with advanced ALK-rearranged NSCLC, including patients with CNS progression and those who had received local therapy.

## MATERIALS AND METHODS

Data on patients with locally advanced or metastatic ALK-positive NSCLC who were treated at the Department of Medical Oncology, Sun Yat-Sen University Cancer Center during the period January 2012 to July 2015 were collected and analyzed retrospectively. The research was approved by Ethics Committee of Sun Yat-Sen University Cancer Center.

ALK-rearrangements were detected by fluorescence in situ hybridization (FISH) analysis or the Ventana ALK(D5F3) immunohistochemical (IHC) test.

### Treatments and outcomes evaluated

All patients received crizotinib therapy which was given in a starting dose of 250 mg twice daily, with appropriate dosing modification if necessary. Clinical characteristics such as the patients’ treatment history, initial response to crizotinib, time to PD, site of PD, and the duration of continued crizotinb and locoregional therapy beyond PD were recorded. Continued crizotinib therapy was defined as >3 weeks of treatment beyond PD, which was defined according to version 1.1 of the Response Evaluation Criteria in Solid Tumors (RECIST) [10]. Additional local therapy was also given depending on the physicians’ assessment of the patients’ symptoms and radiological data.

Outcomes evaluated included the initial and post-initial PFS times (PFS1 and PFS2, respectively). PFS1 was defined as the time from the initiation of crizotinib treatment to the first radiological evidence of PD. Patients whose best response to initial crizotinib therapy was PD were not included in the analysis, as these patients would derive little or no benefit from continued crizotinib therapy [9]. PFS2 was defined as the time from the first to the second RECIST-defined PD with continued crizotinib therapy, or to the discontinuation of crizotinib or a physician-determined change in its usage. Patients without a second documented occurrence of disease progression or who died from any cause were censored on the date of the last follow-up. Follow-ups and the collection of clinical data ceased on January 21, 2016.

### Statistical analysis

The Kaplan-Meier method was used to estimate PFS1 and PFS2. The statistical significance of survival differences between patient groups was tested by log-rank analysis. Cox proportional hazards model analysis was used to screen independent predictive factors for patient survival. Statistical significance was defined as a two-sided p value less than 0.05. All statistical analyses were conducted with SPSS^®^ software, version 22.0 (SPSS Inc, Chicago, IL, USA).

## Results

### Patient characteristics

Among the patients with ALK-positive NSCLC treated at our institution, 33 received continued crizotinib therapy after RECIST-defined disease progression. The patients’ median age was 46 years (range, 21-68 years); 20 (60.6%) were male and 13 (39.4%) were female (Table 1). Ten patients (30.3%) were current smokers, while 23 were either never smokers or had no smoking history. All 33 patients were diagnosed pathologically as having adenocarcinoma; 10 (30.3%) tested by Ventana ALK(D5F3)-positive and 23 (69.7%) tested by FISH-positive. Ten patients (30.3%) were therapy-naïve, while 23 (69.7%) received crizotinb as either second- or third (or greater)-line therapy. The median follow-up time for the 33 patients studied was 17.6 months (range, 8.1-46.1 months).

**Table 1:** Characteristics of the patients who received crizotinib therapy beyond PD (CBPD)

Characteristic	*n* (%)^a^
Age:	
<65 years	31 (93.9%)
≥65 years	2 (6.1%)
Mean (range), years	46 (21-68)
Sex:	
Male	20 (60.6%)
Female	13 (39.4%)
Smoking history:	
Current smoker	10 (30.3%)
Never smoked	21 (63.6%)
Unknown	2 (6.1%)
Histology:	
Adenocarcinoma	33 (100%)
Others	0 (0%)
*ALK* testing method:	
FISH	23 (69.7%)
Ventana ALK(D5F3)	10 (30.3%)
Prior lines of treatments before crizotinib:	
One	14 (42.4%)
Two or more	9 (27.3%)
None (therapy-naïve)	10 (30.3%)
Brain assessments before TKI therapy:	
Brain metastasis-positive	15 (45.4%)
Brain metastasis-negative	9 (27.3%)
None	9 (27.3%)
Disease progression metastasis site:	
Lung and pleura	7 (21.2%)
Brain	20 (60.6%)
Bone	3 (9.1%)
Liver	4 (12.1%)
Kidney or adrenal gland	4 (12.1%)

*ALK*, anaplastic lymphoma kinase; CBPD, crizotinib beyond progressive disease; FISH, fluorescence *in situ* hybridization; TKI, tyrosine kinase inhibitor.

The most common sites of disease progression were the brain (20 patients; 60.6%), followed by the lung, kidney or adrenal gland, and the liver (Table 1).

### Efficacy of initial crizotinib therapy

**Figure 1:**
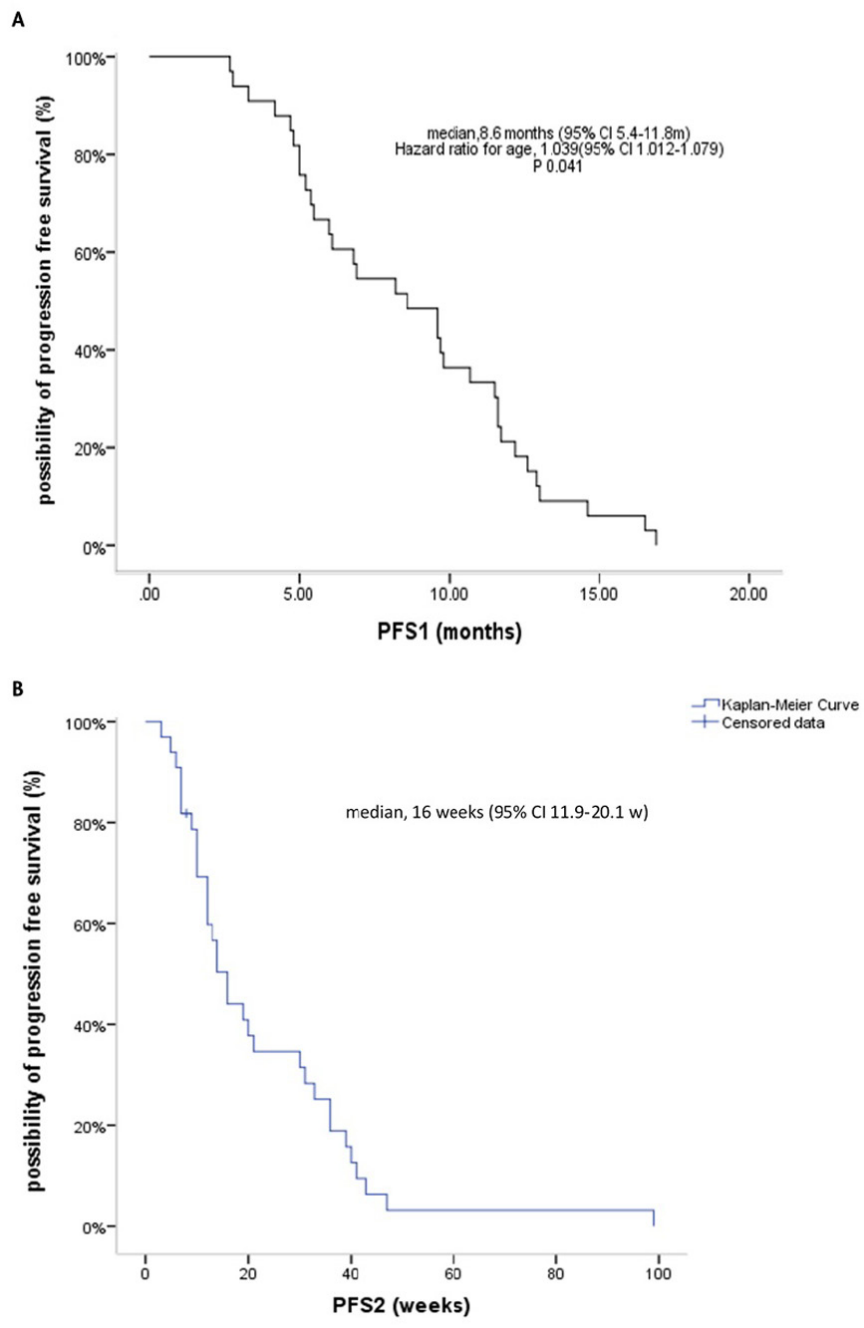
Kaplan-Meier curves for PFS1 with initial crizotinib therapy before progressive disease (PD) (A), and PFS2 with continuation of crizotinib therapy beyond PD (**B**).

The ORR with initial crizotinb therapy was 63.6% (95% CI, 47.2%-80.1%), with 21 patients having a partial response (PR) and the remainder having either tumor control or stable disease (SD); the latter included 2 patients who had no baseline radiological data but exhibited tumor shrinkage in a follow-up computed tomography (CT) scan. The median PFS1 with initial crizotinb treatment was 8.6 months (95% CI, 5.4-11.8 months); 21 patients (63.6%) received crizotinib therapy for 6 months and 6 (18.2%) received it for 12 months before disease progression. Multivariable Cox regression analysis showed that age was the only independent predictor of PFS1 (HR 1.039, 95% CI, 1.002-1.079; *P* = 0.041) (Figure 1A), but the age's groups were incomparably in baseline.

### Brain assessments during crizotinib therapy

Brain assessments were performed by magnetic resonance imaging (MRI) or CT scans in 24 patients (72.7%) within 3 months before the initiation of crizotinb treatment; 15 patients (45.4%) were found to be brain metastasis (BM)-positive, while 9 (27.3%) were BM-negative. Before starting crizotinib therapy, most of the BM-positive patients (10/15, 66.7%) had been treated with local therapy, including brain surgery, whole-brain radiation therapy (WBRT), or stereotactic body radiation therapy (SBRT), and these patients had a longer PFS1 than others (*n* = 5) who did not receive local brain therapy (9.6 months *vs* 4.8 months, respectively).

In patients with brain metastases, brain scans were performed every 2 to 6 months, but other patients had brain scans less frequently, usually 6-monthly or annually. It was remarkable to find that brain progression occurred in 20 of the 33 patients (60.6%). Among the 24 patients who had initial brain assessments, CNS progression occurred in 13 of 15 (86.7%) patients who were initially BM-positive, as compared with 4 of 9 (44.4%) who were initially BM-negative. Even so, stable disease was achieved with continued crizotinib therapy with a median PFS2 time of 14 weeks in the 20 patients with CNS progression (including patients with only CNS disease progression and those with both CNS and extra-CNS progression), and 30 weeks in patients who had CNS progression only and systemic disease control (*n* = 15).

**Table 2:** Clinical outcome of treatment of *ALK*-positive patients with brain metastases

Brain assessment before TKI therapy (*n* = 33)	PFS1 (months)	Patients with CNS progression (*n* = 20)	PFS2 (weeks)
BM-positive (*n* = 15; 45.4%)	8.2^b^	BM-positive (*n* = 13; 65.0%)	14 (patients with CNS disease progression ± extra-CNS progression; *n* = 20) 30 (patients with disease progression only in the CNS; *n* = 15)
Local therapy (*n* = 10)	9.6		
No treatment (*n* = 5)	4.8		
BM-negative (*n* = 9; 27.3%)	9.6^b^	BM-negative (*n* = 4; 20.0%)	
Unknown (*n* = 9; 27.3%)	6.8	Unknown (*n* = 3; 15.0%)	

^b^The difference in PFS1 between BM-positive and BM-negative patients was not statistically significant (*P* = 0.496).

BM, brain metastasis; CNS, central nervous system; PFS, progression-free survival; PD, progressive disease.

### Efficacy of crizotinib therapy continued beyond PD

**Figure 2:**
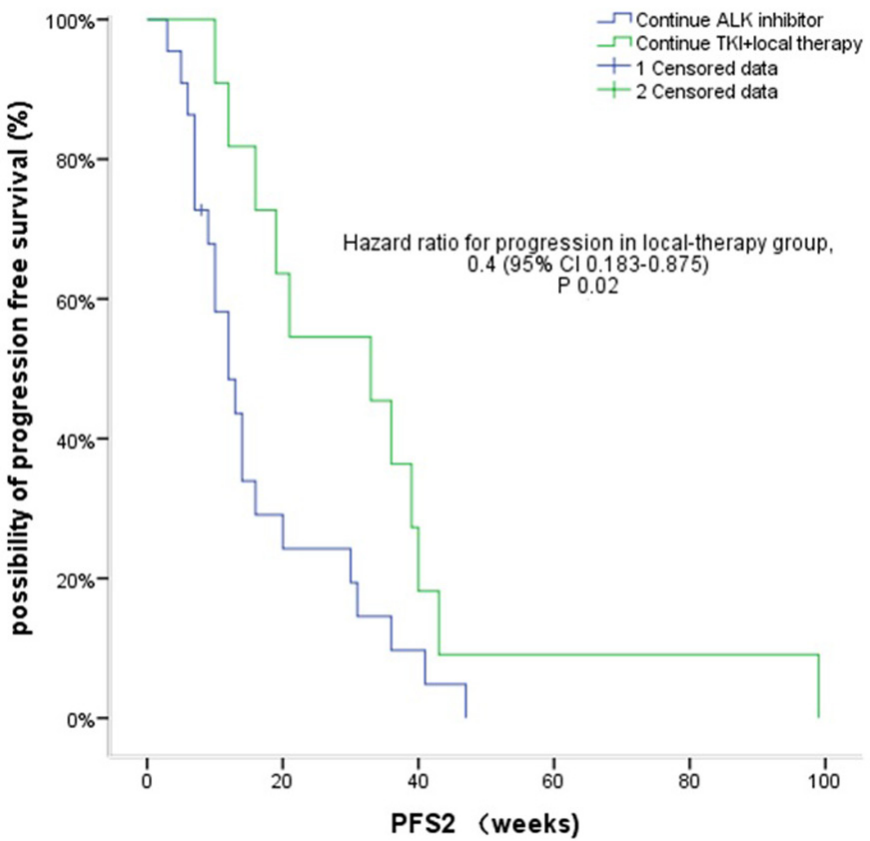
Kaplan-Meier curves for PFS2 in patients who received local therapy versus those who did not

The median PFS2 with continued crizotinib therapy beyond PD in the 33 patients studied was 16 weeks (95% CI, 11.9-20.1 weeks) (Figure 1B). The DCR rate is 75.7% during CBPD with 25 patients had disease control. Cox regression analysis showed that after correcting for confounding factors, PFS1 (HR 1.113, 95% CI, 1.018-1.217; *P* = 0.019) and local therapy (HR 0.4, 95% CI, 0.183-0.875; *P* = 0.022) were independent prognostic factors for PFS2. In 11 patients (33.3%) who received local therapy, the median time from first evidence of radiological progression to initiation of local therapy was 11.6 weeks. Two patients with bone progression received palliative radiotherapy, 1 with both new brain growth and adrenal metastasis received adrenal ablation and seed implantation, 1 had liver cryoablation, and 7 were treated with brain radiotherapy. In this group of patients, the PFS2 was 33 weeks (95% CI, 14.7-51.3 weeks) which was significantly longer than the PFS2 of the 22 patients who did not receive any local treatment (12 weeks, 95% CI, 7.6-16.4 weeks; *P* = 0.039) (Figure 2).

**Figure 3:**
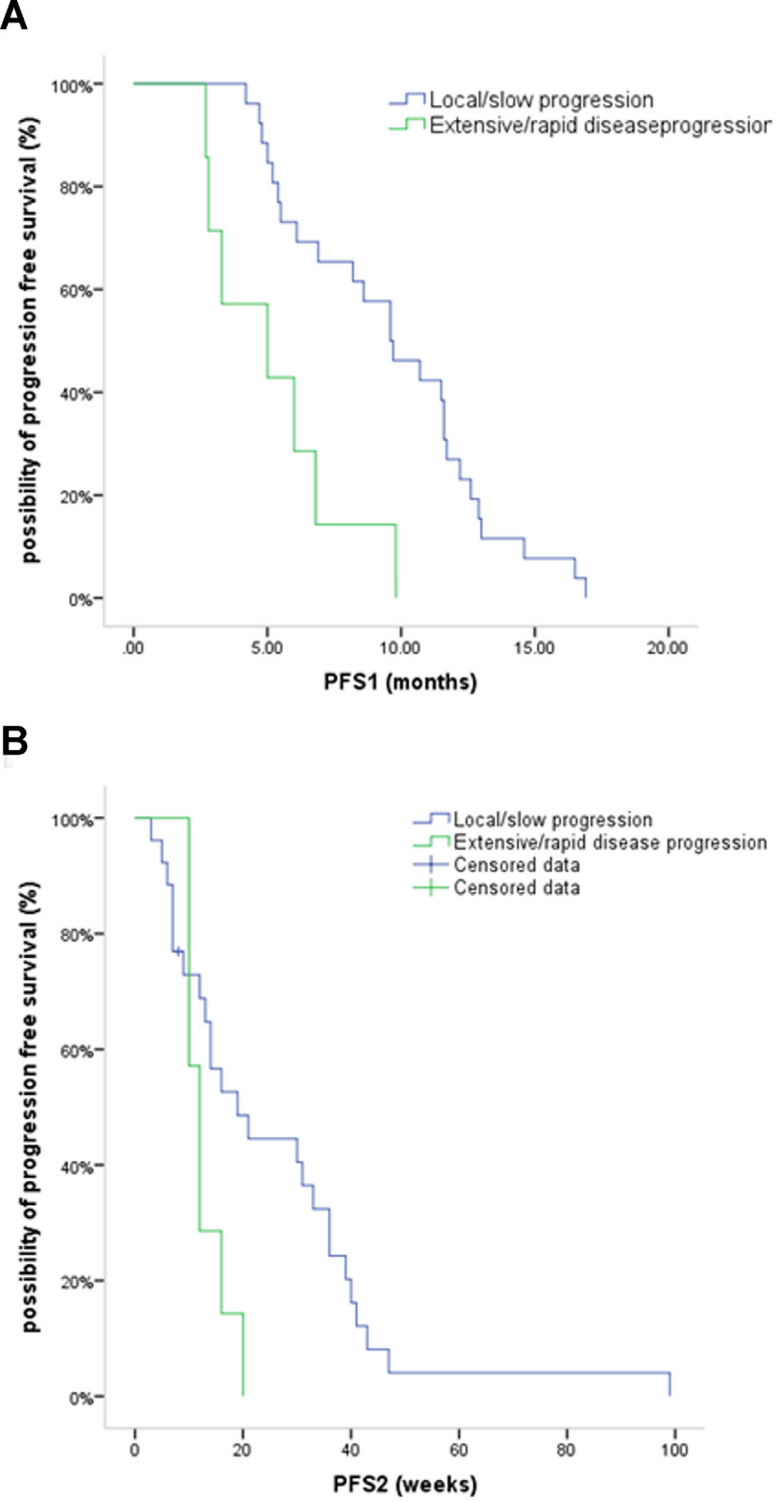
Kaplan-Meier curves for PFS1 (**A**) and PFS2 (**B**) in patients who had extensive/rapid disease progression versus those with local/slow progression.

Among patients who did not receive local therapy, 4 had rapidly progressive disease on continued crizotinb treatment, as did 3 of those who received local therapy. The median PFS1 time for these 7 patients with extensive/rapid disease progression was significantly shorter than the median PFS1 time for the remaining 26 patients who had local/slow progression. Similarly as the PFS2 survival time (12 weeks, 95% CI, 9.6-14.3 weeks *vs* 19 weeks, 95% CI, 7.7-30.3 weeks, respectively; *P* = 0.05), although the 95% CI values overlapped (Figure 3).

## DISCUSSION

In this study of 33 patients who received continued crizotinib therapy after RECIST-defined disease progression, the ORR and initial PFS time (PFS1) were consistent with those reported in phase 1-3 clinical trials of crizotinb monotherapy in patients with ALK-positive lung cancer [7, 8, 11]. However, despite its effectiveness in these patients, disease progression eventually occurs. Especially in patients who harbor gene alterations, continuation of initial TKI therapy is now routinely used in clinical practice [8, 9, 12, 13]. In our study, continuation of crizotinib therapy achieved a median post-initial PFS time (PFS2) of 16 weeks. As a multivariate analysis showed that the PFS1 time and local therapy were independent predictors of the PFS2 time, patients with a longer PFS1 and those who receive local therapy for disease progression may have longer period of continuing crizotinib therapy without changing systemic treatment. This finding is consistent with the results of a previous retrospective analysis of 120 patients with ALK-positive NSCLC which reported a survival benefit with crizotinib treatment continued for a median period of 19.4 weeks beyond disease progression [9].

CNS progression is often the first indication of acquired resistance to crizotinib in patients with ALK-positive NSCLC. Although a CNS response has been reported with crizotinib therapy [14, 15], the CNS has been reported to be a frequent site of initial crizotinib failure in case studies and small retrospective analyses [16–18]. The relatively poor CNS penetration of crizotinib has been suggested to be the underlying mechanism of treatment failure in the CNS as the CSF-to-plasma ratio of crizotinib is low (0.0026) such that brain metastasis are commonly seen despite control of systemic disease [17]. In our study, 20 patients (60.6%) had brain progression during initial crizotinib therapy (15 of whom had isolated CNS progression only), and CNS progression was much more frequent in patients who were BM-positive before crizotinib therapy than those were previously BM-negative (86.6% *vs* 38.8%, respectively). Similar values were reported in a pooled analysis of data from the PROFILE 1005 and 1007 studies [14]. Several studies examining CNS progression during crizotinib therapy have recommended the resumption of treatment with TKIs after radiotherapy [19, 20]. 14 patients in our study had received local brain therapy before or during crizotinib treatment, the median PFS2 time was 3 months in those with CNS progression ± extra-CNS progression, and 8 months in those with only CNS progression. As radiotherapy can control brain tumors and improve CNS symptoms rapidly, and chemotherapy agents are often terminated for brain metastases in patients with NSCLC [21], continuation of both crizotinib and local therapy may contribute to disease control in patients with CNS progression during crizotinib treatment.

Analysis of the progression pattern in our patients (Table 3) indicates the occurrence of acquired resistance to crizotinib. The majority of patients in the present study who had disease progression at local sites (22/33; 66.7%) had minimal progression or were asymptomatic, and local therapy with continued crizotinib was beneficial in prolonging the PFS2 time in these patients. Weickhardt et al.[20] hypothesized that under selective pressure, multiple drug resistance mechanisms and tumor biology alterations occur stochastically favoring survival in accordance with Darwinian evolutionary principles, and that local therapy could help in controlling resistant mutations before their expression results in systemic disease progression. These authors suggested that patients suitable for local therapy are those with either non-leptomeningeal CNS involvement and/or ≤4 extra-CNS sites, and that continuation of the targeted agent in these patients is associated additional disease control. For patients with CNS progression, this treatment model is especially recommended if systemic disease is responding or is stable with initial crizotinb therapy, which was seen in 12/22 (54.5%) patients with brain progression in our study. Some studies have reported that local ablative approaches and continued crizotinb therapy can prolong disease control by 5.5-10 months [19, 20, 22, 23], and one study [23] identified EML4-ALK translocations as radiosensitive genotypes for which superior control rates are obtained with radiotherapy. As improvement of PFS2 with the available local therapies is inconsistent, and there are currently only limited data on their safety and survival benefits, we did not utilize defined criteria for their administration. Further prospective studies are needed to evaluate the benefit of local therapy plus crizotinib combinations. In the 7 patients in our study who had extensive/rapid disease progression with shorter PFS1 and PFS2 times (Figure 3), the median overall survival was 24 weeks. These patients exhibited little benefit from continued crizotinib therapy, and in such cases other chemotherapy regimens should be considered.

**Table 3:** Progression pattern of 24 ALK-positive NSCLC patients with crizotinib therapy

Patient No.	Brain metastasis (BM) status before crizotinib	Local therapy before crizotinib	PFS1 with crizotinib (mths)	Site of disease progression	Progression pattern	Treatment for disease progression	PFS2 (wks)
1	BM-negative	None	16.9	Brain	New lesion	CBPD	7
2	BM	None	4.8	Bilateral renal	New lesion	CBPD	41
3	BM	None	2.8	Brain	New lesion	CBPD	12
4	BM	Brain surgery and SBRT	5.4	Brain	New lesion	CBPD	36
5	BM-negative	None	14.6	Brain and lymph node	New brain lesion and lymph node regrowth	CBPD	3
6	BM	SBRT	5.5	Brain	Regrowth	CBPD	13
7	BM	Radiotherapy	9.6	Brain and multiple pulmonary nodules	New brain lesion and pulmonary regrowth	CBPD	6
8	BM	SBRT	16.5	Brain	Regrowth	CBPD	30
9	BM-negative	None	11.7	Brain	New lesion	CBPD	9
10	BM	None	4.7	Brain	Regrowth	CBPD	14
11	BM	WBRT	9.7	Brain	Regrowth	CBPD	8
12	BM	SBRT	12.9	Lung	Regrowth	CBPD	7
13	BM	None	11.6	Brain	New lesion	CBPD	14
14	BM	SBRT	11.6	Brain	New lesion	SBRT	33
15	Unknown	None	5.2	Brain	New lesion	WBRT + SBRT	36
16	BM-negative	None	4.2	Brain	New lesion	WBRT	99
17	Unknown	None	9.8	Brain and bone	Regrowth	SBRT (bone)	12
18	BM	SBRT	13.0	Brain	Regrowth	SBRT	40
19	BM	WBRT	8.2	Brain	New growth	SBRT	43
20	BM	WBRT	3.3	Brain and adrenal	New brain lesion and adrenal regrowth	Adrenal ablation & seed implantation	10
21	BM	None	6.1	Brain	Regrowth	SBRT	39
22	Unknown	None	12.6	Brain	New lesion	SBRT	19

BM, brain metastasis; CBPD, crizotinib continued beyond progressive disease; NSCLC, non—small-cell lung cancer; PFS, progression-free survival; SBRT, stereotactic body radiation therapy; WBRT, whole-brain radiation therapy.

The mechanism of acquired resistance to crizotinib treatment is heterogeneous. Several mechanisms have been reported, including generation of secondary resistant mutations, amplification of the ALK gene, and activation of other bypass signaling pathways [24–26], which suggests that treatment after disease progression is complex. Changes of oncogenes lead to altered sensitivity to crizotinb and possibly to second-generation ALK inhibitors such as ceritinib [27]. While continuing TKI therapy still controll existing sensitive tumor cells, disease flare can be seen in patients who discontinue TKI treatment [28, 29]. Recently, NCCN guide-line have recommended the second-generation ALK inhibitors ceritinib and alectinib for the treatment of patients with acquired resistance to crizotinib [30–32], but these agents are not yet available for use in china. Our studies provide a feasible strategy as continuing crizotinib after disease progression.

At the date of the last follow-up, 17 of our patients who continued on crizotinib therapy had not experienced a second disease progression, and overall survival was unable to be determined. The impact of continuing crizotinib to OS is indefinitely, therefore clinical benefit resulting from delaying chemotherapy could avoid more side effects and relieving financial pressure of other systemic therapy, especially the next-generation ALK-inhibitors.

In conclusion, this study, which was derived from real-world clinical experience in China, provides additional evidence of the efficacy of continuing crizotinib therapy after disease progression in patients with advanced ALK-positive NSCLC. The main limitation of our study, namely, the small number of patients studied, and highlights the need for further studies to investigate the mechanism of disease progression and the efficacy of treatments administered after the second disease progression.
